# False-positive urine pregnancy tests - clinicians as detectives

**DOI:** 10.4314/pamj.v8i1.71156

**Published:** 2011-04-10

**Authors:** Rolando Valenzuela, Kenneth V Iserson, Damien Punguyire

**Affiliations:** 1 Department of Emergency Medicine, University of Medicine and Dentistry of New Jersey, Newark, New Jersey, USA; 2 Department of Emergency Medicine, The University of Arizona, 4930 N. Calle Faja, Tucson, AZ 85718, USA; 3 Kintampo Municipal Hospital Kintampo, Ghana

**Keywords:** Urine pregnancy test, false-positive tests, laboratory tests

## Abstract

Reliably diagnosing pregnancy in women presenting with nonspecific abdominal pain can be lifesaving. If diagnostic tests are unreliable, however, valuable time and resources can be wasted pursuing unnecessary and potentially harmful interventions. After four false positive-urine pregnancy tests in one week, we began investigating the laboratory’s entire process involving the UPreg tests. We discovered that, as is common in resource-poor settings, the laboratory repeatedly reused test tubes. We found that the false-positive tests resulted from performing the UPreg tests in test tubes that were improperly cleaned and, for the most part, had been used immediately beforehand to test women coming into the maternity ward. Sufficient residua from the pregnant women’s high ß-HCG levels had remained in the test tubes to cause subsequent false-positive results in our emergency ward patients. Although pregnancy can now be reliably diagnosed with inexpensive, disposable and simple tests, these tests must not only be used properly, but also, when used in the laboratory, be accompanied by appropriate cleaning and quality-control procedures. This is particularly essential in resource-constrained environments.

## Background

Reliably diagnosing pregnancy in women presenting with nonspecific abdominal pain can be lifesaving. If diagnostic tests are unreliable, however, valuable time and resources can be wasted pursuing unnecessary and potentially harmful interventions. Discovering the reason why relatively simple laboratory tests are unreliable evokes the spirit of Sherlock Holmes (aka, Dr. Joseph Bell).

For millennia, clinicians have used urine tests to verify pregnancies. As far back as 1350 B.C., Egyptians had women urinate on a mixture of wheat and barley seeds, believing that sprouting seedlings would indicate pregnancy (Verification tests in the 1960s found accuracy approaching 70%!). In the middle-age, “piss-prophets” claimed that they could predict pregnancy using the color of a woman’s urine, while in modern times, importance was placed on body temperature, the medical history and the physical examination [1]. These methods, though widely used, were far less reliable than those of the ancient Egyptians.

Several relatively accurate, but expensive and time-consuming pregnancy tests became available in the 1950s and 1960s. In 1976, disposable qualitative immunological assays using antibodies to ß-HCG [2] appeared, bringing a higher level of accuracy and, eventually, more rapid and inexpensive tests. Modern urine pregnancy point-of-care tests (UPreg POCT) have a sensitivity >99.5% for detecting ß-HCG at levels P25 IU/L [3]. Used in clinics, laboratories, homes, and Emergency Departments, the UPreg POCT test is often the sole method available to diagnose early pregnancy in resource-constrained settings.

While working at the Kintampo Municipal Hospital Emergency Ward in central Ghana, we relied on urine pregnancy tests, a menstrual history (often sketchy), and the physical exam as the sole means to emergently determine pregnancy in women presenting with abdominal pain. We observed several patients with positive laboratory-performed UPreg test who adamantly denied they could be pregnant. Repeated UPreg tests confirmed that, indeed, they were not pregnant.

## Methods

After four false positive episodes in four different women of reproductive age in the course of one week, we began investigating the laboratory’s entire process involving the UPreg tests (The UPreg test kits are generic and have an unidentifiable Chinese manufacturer). Patient’s urine samples were taken to the lab in disposable plastic containers to our laboratory in a separate building. Laboratory technicians transferred the urine to glass test-tubes, from which they withdrew small samples via pipette. Several drops of urine were then placed on individual pre-packaged one-time use ß-HCG Urine Pregnancy POCT kits of Chinese manufacture, where two lines indicate pregnancy, and one line indicates the absence of pregnancy.

## Results

We discovered that, as is common in resource-poor settings, the laboratory was reusing test tubes. We found that the false-positive tests resulted from performing the UPreg tests in test tubes that were improperly cleaned and had been used immediately beforehand to test women coming into the maternity ward. Due to a limited supply of test tubes, the lab routinely reused them when they thought it did not alter test results. In these cases, sufficient residua from the pregnant women’s high ß-HCG levels had remained in the test tubes to cause subsequent false-positive results in the Emergency Ward patients. When these patients were tested using clean test tubes, their ß-HCG Urine Pregnancy test results were negative.

## Conclusion

Pregnancy can now be reliably diagnosed with inexpensive, disposable, and simple tests. This test is invaluable because of its ease of use and accuracy; in fact, they often seem foolproof. However, it is essential that it be used properly and - when used in the laboratory - accompanied by rigorous cleaning procedures and regular quality-control evaluations. This is crucial in resource-poor environments where few, if any, tests may be available to confirm or refute an incorrect result. Our experience showed that even this simple test can be made frequently inaccurate if these standards are not followed. This can result in false positives which can lead to erroneous diagnosis, unnecessary procedures, and potentially dangerous outcomes. As in our case, if laboratory results don’t match the clinical picture, repeat the tests. If the situation recurs, follow Sherlock Holmes’ lead and investigate.

## Competing interests

The authors declare no competing interests.

## Authors’ contribution

All authors have equally contributed to the write-up of the manuscript and have read and approve the final version.
